# A novel molecular rotor facilitates detection of p53-DNA interactions using the Fluorescent Intercalator Displacement Assay

**DOI:** 10.1038/s41598-018-31197-9

**Published:** 2018-08-28

**Authors:** Walter L. Goh, Min Yen Lee, Ting Xiang Lim, Joy S. Chua, Sydney Brenner, Farid J. Ghadessy, Yin Nah Teo

**Affiliations:** 1 Molecular Engineering Lab, Biomedical Sciences Institutes, A*STAR, 61 Biopolis Drive, Singapore, 138673 Singapore; 2 p53 Laboratory, A*STAR, 8A Biomedical Grove, #06-06 Immunos, Singapore, 138648 Singapore

## Abstract

We have investigated the use of fluorescent molecular rotors as probes for detection of p53 binding to DNA. These are a class of fluorophores that undergo twisted intramolecular charge transfer (TICT). They are non-fluorescent in a freely rotating conformation and experience a fluorescence increase when restricted in the planar conformation. We hypothesized that intercalation of a molecular rotor between DNA base pairs would result in a fluorescence turn-on signal. Upon displacement by a DNA binding protein, measurable loss of signal would facilitate use of the molecular rotor in the fluorescent intercalator displacement (FID) assay. A panel of probes was interrogated using the well-established p53 model system across various DNA response elements. A novel, readily synthesizable molecular rotor incorporating an acridine orange DNA intercalating group (AO-R) outperformed other conventional dyes in the FID assay. It enabled relative measurement of p53 sequence-specific DNA interactions and study of the dominant-negative effects of cancer-associated p53 mutants. In a further application, AO-R also proved useful for staining apoptotic cells in live zebrafish embryos.

## Introduction

Interactions between proteins and DNA are essential cellular processes. Examples of DNA transacting proteins include transcription factors, polymerases, telomerases as well as factors involved in DNA repair pathways. Compromised protein-DNA interactions can give rise to severe disease phenotypes, notably cancer^1^. There exists therefore, a requirement for robust assays enabling fundamental understanding of molecular interactions, high-throughput identification of compounds that restore correct DNA-binding in compromised cellular targets, and assessment of DNA binding proteins in clinical diagnostics^2^. The p53 protein is a tumor suppressor crucial in preventing cancer through the maintenance of cellular homeostasis and genomic integrity^3^. It senses intracellular disturbances, particularly those that promote tumorigenesis (radiation damage, hypoxia, glucose starvation, oncogene activation), and functions to limit damage by augmenting sophisticated cellular responses that include cell-cycle arrest, DNA repair, apoptosis and cellular senescence^3^. As a transcription factor, p53 recognizes and binds cognate DNA elements to transcriptionally regulate a plethora of gene targets. Mutations in p53 have been found in more than half of human cancers^4^ and typically result in the translation of full-length mutant proteins defective in DNA binding.

Traditional methods for determining protein-DNA binding include the electrophoretic mobility shift assay (EMSA)^5^ and DNA footprinting^6^. These methods are technically demanding, have limited sensitivity and throughput, and often require the use of radioisotopes. A safer, more facile alternative has been developed based on the ELISA format^7^, and more recent variations include using flow analysis on fluorescently-labelled microspheres^8^, and bead-based microscopy imaging^9^. Other techniques involving surface plasmon resonance (SPR)^10^, protein induced fluorescence enhancement (PIFE)^11^, and quantitative-PCR^12^ are powerful but require expensive instrumentation or synthetic labels, and may not be suited for high-throughput applications. Fluorescence anisotropy measurement is a widely adopted method for fast, accurate analysis of DNA-protein binding^13–15^. Whilst undoubtedly facile, it requires defined labeling of each target DNA, potentially limiting its use in higher throughput screening campaigns interrogating protein binding to larger DNA libraries.

The availability of a homogenous, low volume, non-radioactive assay with fluorescent readout will benefit both academia and industry. The fluorescent intercalation displacement (FID) assay meets many of these criteria in addition to being cost-effective, facile and robust. FID measures binding of ligands to DNA through the displacement of a fluorogenic compound (e.g. ethidium bromide and thiazole orange) pre-bound to DNA, resulting in a decrease in fluorescence intensity^16^. These features have led to development of high-throughput applications in combination with cognate site identification (CSI-FID) in a microarray platform to examine netropsin-DNA interaction^17^. Whilst the FID assay has been used to evaluate binding of numerous compounds, there have only been few reports of its use with proteins^17–21^. Herein, we explore the utility of a novel class of fluorescent compounds known as molecular rotors for detection of p53 binding to DNA in assays modeled after the FID phenomenon.

Molecular rotors are a collective group of fluorescent compounds that possess the ability to undergo twisted intramolecular charge transfer (TICT)^22^. They typically consist of three parts: an electron-donating unit, an electron-accepting unit and a π-conjugated linking moiety which allows electron transfer to occur in the planar conformation. Upon irradiation, electrostatic forces result in the molecule adopting a twisted conformation around the σ-bond in the linker region. This non-planar, twisted conformation has a lower excited state energy, and is thus associated with either a red-shifted fluorescence emission or a non-radiative torsional relaxation pathway, depending on the molecular structure of the rotor. The TICT properties of molecular rotors are highly sensitive to their micro-environment. Hindrances in intramolecular rotation, through higher viscosity for instance, prevent the non-radiative pathway and results in fluorescence restoration as the molecule adopts a planar configuration. The inherent sensitivity of molecular rotors to the polarity and viscosity of their environment, allowing for simultaneous fluorescence readouts, makes them highly attractive as fluorescent probes. The julolidine malononitriles have been used as viscosity sensor probes in lipid membranes^23^. Other motifs such as thiophene-containing rotors have also been developed as viscosity sensors^24^. Besides fluorescence emission, the fluorescence lifespan of benzylidene malononitriles have also been used as a quantitative parameter for ratiometric measurements of viscosity in live cells^25,26^. Furthermore, acridizinium molecular rotors have been shown to be sensitive to DNA binding through intercalation^27,28^. Upon intercalating to DNA, the intramolecular rotation of the molecule around the N-phenyl bond is restricted, leading to an increase in fluorescence signal. N-(halogenphenyl)-9-acridizinium ions in the presence of an anionic surfactant are also capable of binding to albumin proteins, giving a 20-fold increase in fluorescence^29,30^. To date, molecular rotors have demonstrated great value as fluorescent probes, granting quantitative readouts upon viscosity changes as well as molecular interactions within biological systems. An earlier study by our group showed that specific interactions between proteins, as well as their antagonism, can be probed by molecular rotors^31^. Here, we report the design of several novel fluorogenic compounds, and explore their utility in FID assays through assessment of their DNA-binding fluorogenic properties. In particular, a new molecular rotor incorporating an acridine orange DNA intercalating group (AO-R) displayed superior performance over conventional dyes in the FID assay, allowing sensitive measurement of p53 sequence-specific DNA interaction.

## Results

### Chemical design and fluorescent properties of molecular rotors

**Figure 1:**
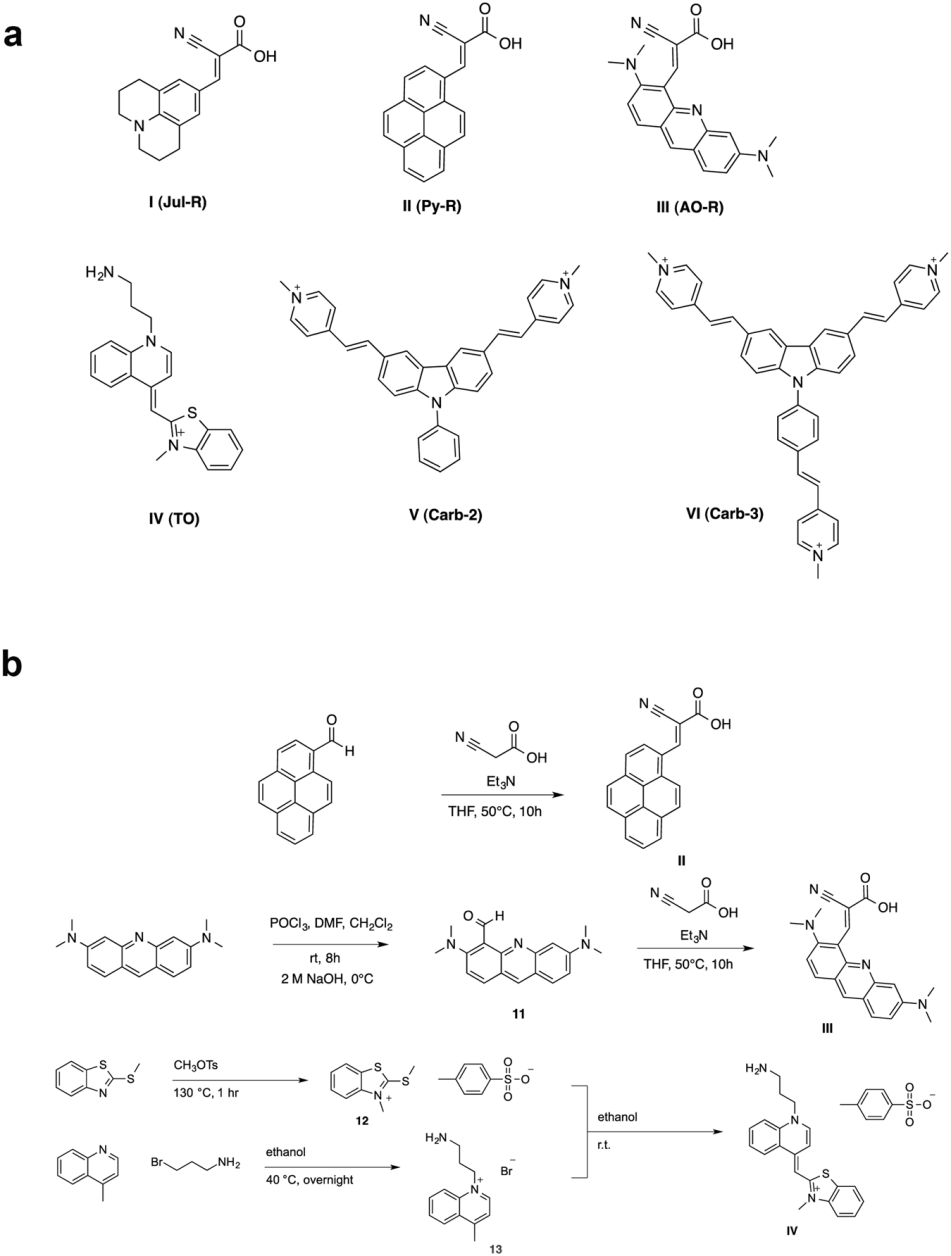
Chemical structures of the molecular rotors used. Shown below are synthetic routes for compounds II, III and IV.

A panel of fluorescent compounds was synthesized and characterized against commonly used fluorescent dyes. 9-(2-carboxy-2-cyano)vinyl-julolidine (CCVJ, Jul-R, Fig. 1) is a previously described molecular rotor which displayed strong TICT in detecting protein-protein interaction^31^. In addition, two new molecular rotors were designed to incorporate a DNA intercalating unit as the electron donor moiety in place of the non-planar tertiary amine of the julolidine motif. The two units chosen, pyrene (Py-R) and acridine orange (AO-R), have flat aromatic structures likely to facilitate intercalation into the DNA helix (Fig. 1, Scheme 1). Utility of these rotors was compared against common probes used in FID assays, ethidium bromide (EtBr) and thiazole orange (TO-C), as well as commercial acridine orange (AO-C). Thiazole orange was also further modified to include a primary amine handle (TO) that TO-C lacks. Furthermore, we studied the use of two minor groove binders with the carbazole motif (Carb-2 and Carb-3) for use in FID (Fig. 1).

The fluorescence intensity of each compound was first examined in solutions with varying viscosities. An increase in viscosity impedes rotation around the bond connecting the two pi-systems, leading to higher structural rigidity and a consequent increase in the fluorescence signal. The extent of fluorescence signal increase relative to the increase in viscosity is an indication of its sensitivity as a molecular sensor. Experiments were performed using ethylene glycol in glycerol where the amount of glycerol was adjusted to vary viscosity. All the rotors synthesized showed an increase in fluorescence upon viscosity increase (Supplementary Fig. 1), demonstrating rotor functionality. Optimal excitation and emission wavelengths recorded were used for further experiments (Table 1).

**Table 1:** Excitation and emission properties of compounds tested.

Compound ID	Compound	Excitation wavelength (nm)	Emission wavelength (nm)
I	Jul-R	433	506
II	Py-R	342, 376	527
III	AO-R	480	526
IV	TO	510	552
V	Carb-2	458	572
VI	Carb-3	456	582

### Characterization of DNA-dependent fluorescence turn-on

**Figure 2:**
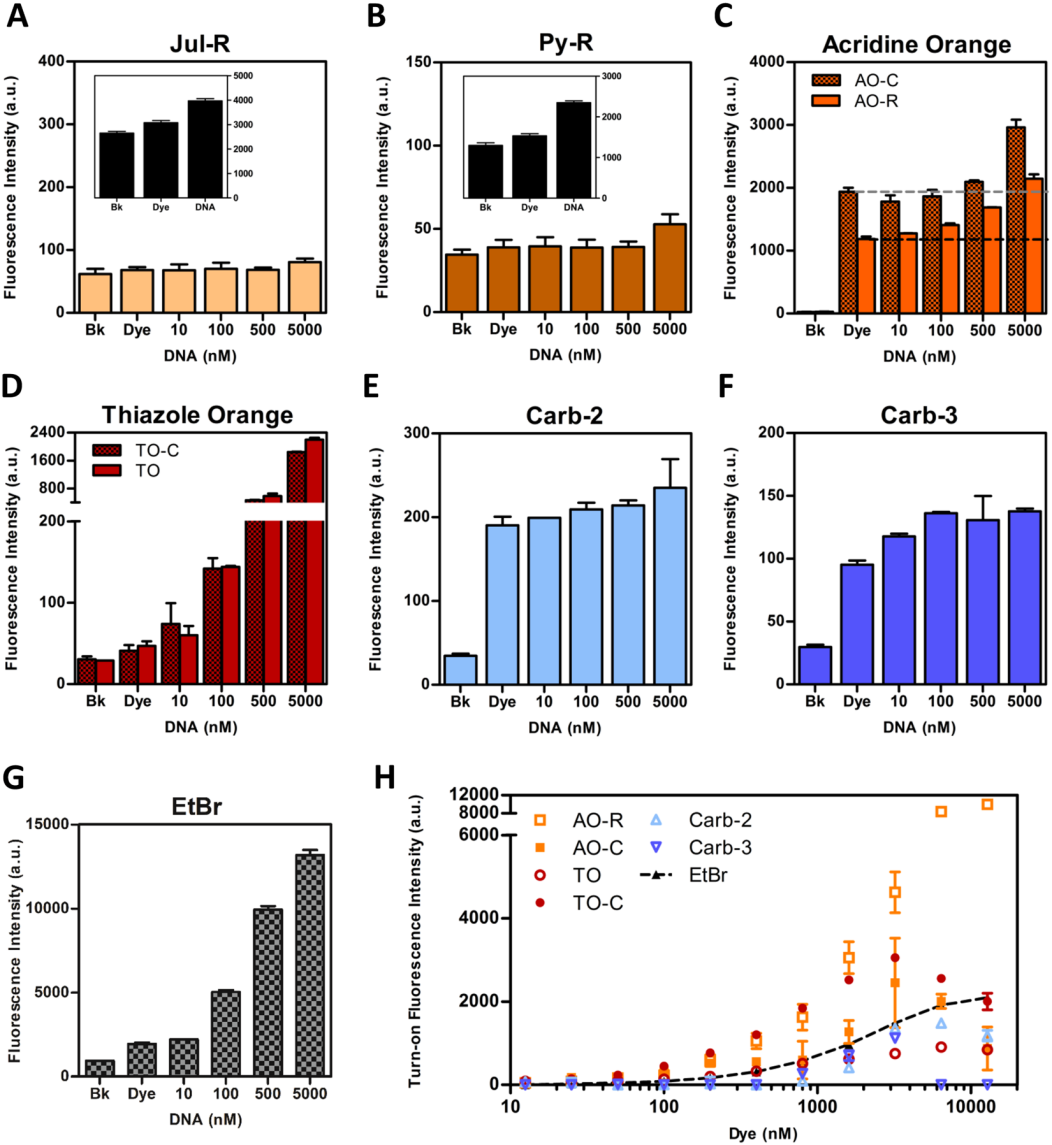
Fluorescence response of molecular rotors in the presence of DNA. (**A**–**G**) Fluorescence signal of rotor molecules at a fixed concentration (100 nM) titrated against increasing concentrations of double-stranded DNA fragments. Black-bar insets show fluorescence signal at maximum measurement configuration and highest DNA concentration. Hashed-lines depict levels of dye-only background fluorescence. (**H**) Levels of turn-on fluorescence from increasing concentrations of each compound in the presence of a fixed concentration of DNA (200 nM). All data shows mean ± S.D. of 3 individual experiments. For Py-R with dual excitation wavelengths, results for 376 nM wavelength shown. Similar results were observed upon excitation at 342 nM (Supplementary Fig. 2).

Increasing concentrations of 30-bp double-stranded oligonucleotides were next added to each compound and induced fluorescence upon DNA binding measured. All synthesized compounds showed variable increases in fluorescence signal in the presence of DNA (Fig. 2). Compounds TO and TO-C showed very similar fluorescence profiles. Notably, AO-R was distinctly more sensitive to DNA-dependent fluorescence turn-on than AO-C, displaying similar levels of signal enhancement when in the presence of 50-fold less DNA (Fig. 2C). AO-R also showed lower background signal and displayed a stronger turn-on signal over AO-C (82% and 52%, respectively, in 5 μM DNA). Compounds TO, TO-C, and AO-R showed fluorescence gains at the lowest concentration of duplex DNA tested (10 nM), equivalent to that observed for the commonly used FID dye ethidium bromide (Fig. 2G). Both minor groove binders, Carb-2 and Carb-3, displayed a gradual but consistent turn-on activity that appeared to saturate in the presence of a relatively lower concentration of duplex DNA (100 nM), possibly indicating stronger binding but comparatively weaker signal activation (Fig. 2E and F). For Jul-R (Fig. 2A) and Py-R (Fig. 2B, Supplementary Fig. 2), notable fluorescence was only apparent at the highest concentration of duplex DNA used (5 µM) (Fig. 2A,B). This may be attributed to topological exclusion arising from the non-planar Jul-R and bulky Py-R, rotor units. The two compounds were deemed unsuitable and excluded from further experiments.

The turn-on signal of each dye was next examined in a reverse configuration by increasing dye concentrations against a fixed amount of DNA (Fig. 2H). DNA-dependent fluorescence enhancement was determined by subtracting dye-only background from total signal in the presence of 200 nM duplex DNA (Supplementary Fig. 3). The EtBr control displayed strong gain, approaching saturation at ~40-fold molar excess over duplex DNA molecules, closely correlating to the expected occupancy of a strong binding intercalator. Both carbazole compounds displayed stronger DNA-induced signal gain than previously observed, which increased with increasing compound concentrations (Supplementary Fig. 3), suggesting optimal activity when present at ~10–15-fold molar excess over DNA molecules. AO-R displayed markedly brighter turn-on signal from DNA binding compared to all dyes, most notably its parental compound, AO-C. In addition, it showed no decline in signal gain at the highest concentration tested, unlike AO-C which displayed diminishing turn-on activity above 3.2 µM dye (16-fold molar excess over duplex DNA molecules). This suggests that fluorescence activation from AO-R is largely DNA-dependent with lesser background signal. Auto-fluorescence was also observed for TO-C, Carb-2 and Carb-3 when used at higher concentrations (Supplementary Fig. 3). In most cases, the phenomenon of background (dye only) signal eclipsing DNA-induced fluorescence was observed, and became increasingly obvious at higher dye concentrations, likely due to off-target interaction or auto-fluorescence from poor solubility.

The p53 tumor suppressor acts as a transcription regulator in cells and mediates gene expression by binding DNA response elements (DNA-RE) carrying the canonical sequence RRRC(A/T) (T/A)GYYY (where R = purine and Y = pyrimidine). Each p53 dimer makes contact with a decamer half-site to form a DNA-tetramer complex^32,33^. Binding of two p53 tetramers to one DNA-RE has also been observed^34^. Further understanding of p53 biochemistry has revealed a high level of degeneracy in DNA recognition^35^, likely evolved from requirements in functional plasticity under varying cellular conditions. In addition to the several hundreds of experimentally verified p53 response elements, thousands more putative sites have been identified in whole genome studies^36–38^. As such, an ideal FID probe for studying highly promiscuous DNA-binding proteins like p53 should possess a strong turn-on signal to afford discernment in DNA selectivity, and also bind DNA with minimal sequence bias to give similar fluorescence baselines across diverse sequences.

**Figure 3:**
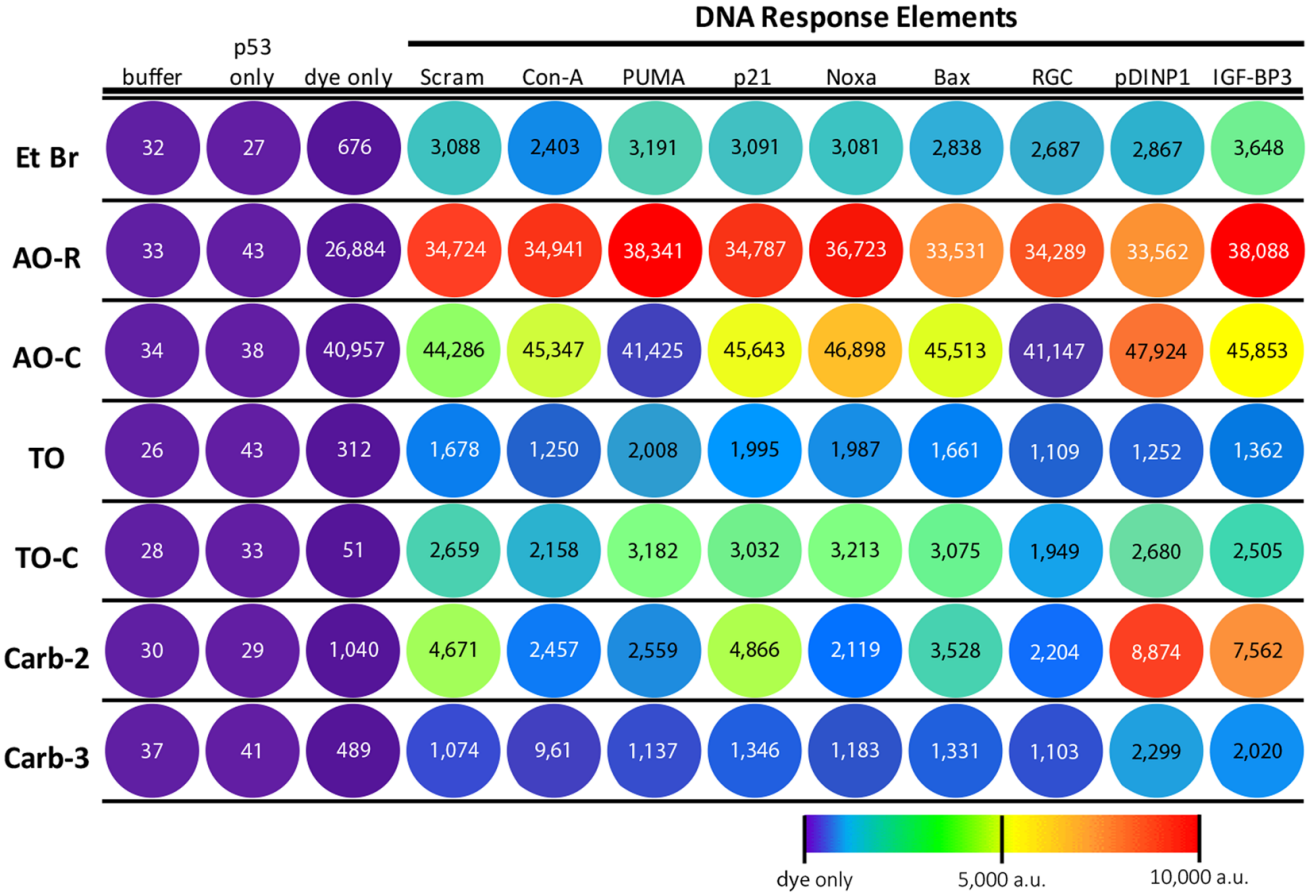
Signal intensity heat map of DNA-induced and sequence-dependent fluorescence intensity across rotor compounds. Color representative heat map showing rotor-specific fluorescence response to a panel of DNA oligonucleotide sequences containing p53 response elements and control sequence (scram). Color spectrum depicts absolute amount of fluorescence gained for each dye in the presence of DNA (color scale bar: violet = self-referenced dye-only signal, red = highest observed Δa.u. across all dyes, set at 10 000 a.u.). Each row depicts fluorescence signals from individual compounds with lower limit (violet) benchmarked to respective “dye only” background signals.

To assess the correlation between DNA sequence and fluorescence intensity, the compounds were tested against a panel of unique DNA sequences comprised of a non-binding scrambled DNA control (Scram) and p53-REs of varying affinities (Supplementary Table 1). A dye concentration of 15-fold molar excess over duplex DNA was used for the purpose of comparative assessment, as it gave the best signal-to-noise ratio for all dyes while being below concentrations that could contribute to aberrant signal readouts observed earlier for certain compounds (Fig. 2H). DNA-induced signals were contrasted across all dyes using a colored heat map to represent fluorescence gain. An ideal rotor would show both a high fluorescence turn-on signal (warm colors in the presence of DNA) and a uniform fluorescence response across different REs. Carbazole compounds displayed the highest degree of signal disparity across DNA sequences. As before, Carb-2 appeared brighter than Carb-3 when DNA-bound, and additionally possessed the potential to be “hyper”-activated in the presence of particular DNA sequences (pDINP1 and IGF-BP3), to which both compounds appear to have an affinity towards (Fig. 3). Both arms of Carb-2 rotor could simultaneously bind within minor grooves of DNA, leading to a brighter response over the 3-arm carbazole, which can still experience significant TICT through a sterically excluded unbound third arm. In contrast, EtBr, TO and TO-C showed more uniform fluorescence across diverse DNA sequences, but only a moderate turn-on fluorescence compared to AO-R, which displayed the highest degree of fluorescence activation and was also sequence-agnostic, especially when contrasted against its commercial variant, AO-C (Fig. 3).

### Molecular rotors as probes for protein-DNA interactions

**Figure 4:**
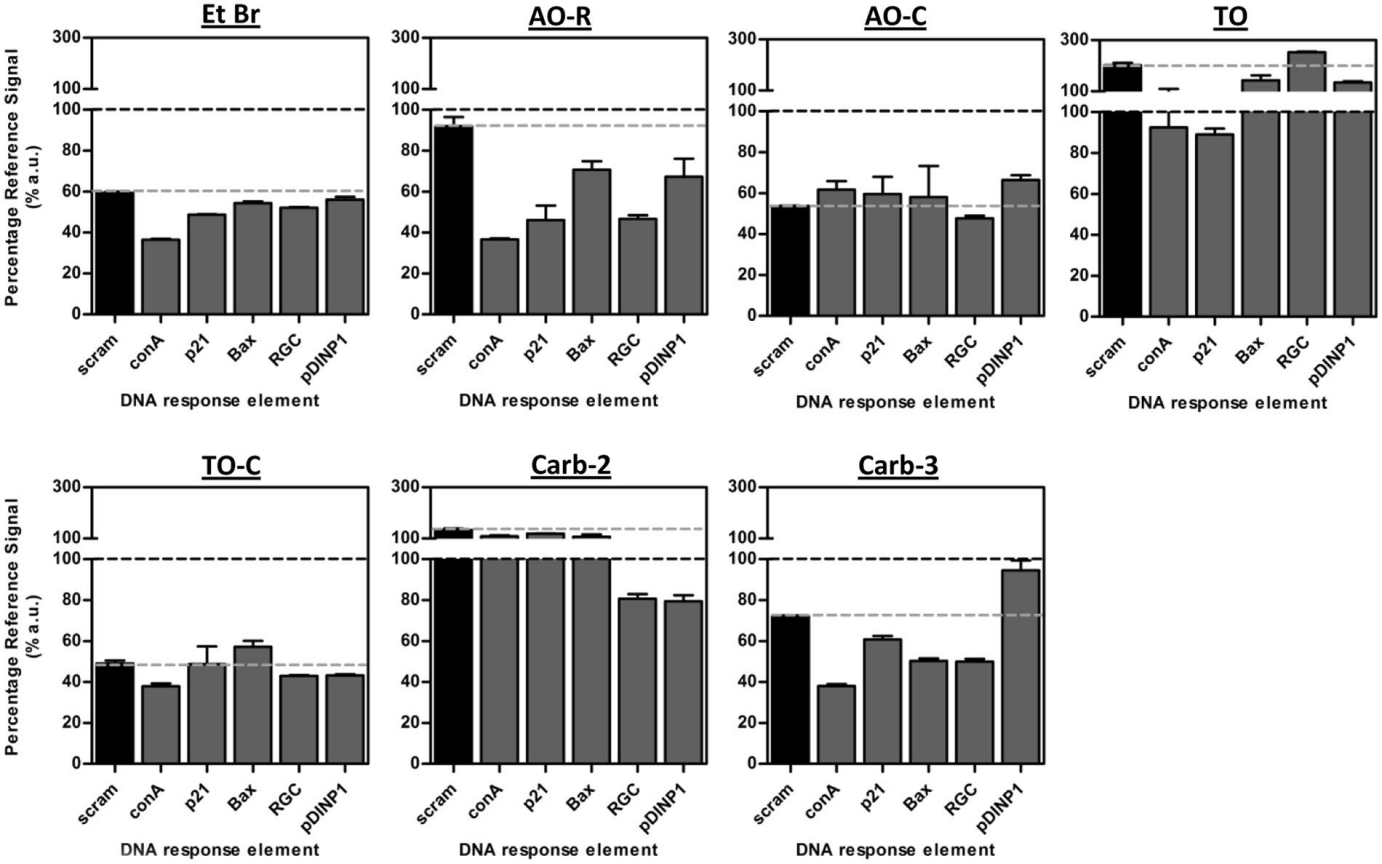
Fluorescent intercalator displacement assay with rotor compounds. Percentage change in fluorescence intensity when purified wildtype-p53 protein is added to mixtures containing rotor pre-complexed with different DNA fragments. Black-hash line depicts 100% reference value (rotor and DNA only), below which shows a displacement event. Grey-hash line shows percentage-change in signal for scrambled-DNA sequence. Data shows mean ± S.D. for 2 biological replicates.

We next investigated the suitability of each compound in an FID assay to measure p53-DNA binding. Purified wildtype p53 protein (p53-WT) was added to specific DNA-REs pre-incubated with rotor compounds. A p53 non-binding sequence (scram) was included as control DNA for non-specific fluorescence attenuation, and together with selected REs known either to bind p53 moderately (Bax, pDINP1) or tightly (conA, p21, RGC), formed the representative panel of DNA response elements. The resultant signal, expressed as a percentage of the reference signal (rotors in the presence of DNA), gives an indication of p53-dependent changes in fluorescence. A displacement event occurs when fluorescence falls below the reference signal in the presence of p53 (black-hashed line, Fig. 4), suggesting liberation of the bulky rotor unit from the intercalating site due to p53-DNA binding, while the scram signal gives a further indication of sequence-specificity.

Compounds AO-C and TO-C displayed sequence-nonspecific decreases in fluorescence whilst TO and Carb-2 showed signal increase (Fig. 4), as seen in the respective signal changes of scram control in the presence of p53. The indiscriminate behavior of these dyes could arise from off-target interaction between dye and p53 molecules, the inability of p53 to recognize or approach DNA motifs due to steric distortions on DNA molecules caused by rotor interaction, or from the sequence-biased turn-on activity discussed earlier. Such factors may be further compounded by solubility issues in aqueous buffers, as noted earlier for several compounds when present at higher concentrations (Fig. 2H) and in thiazole orange compounds (Supplementary Figs 3 and 4). Fluorescence intensity from DNA-RE complexed with Etbr, AO-R and Carb-3 fell below signals from both reference value and scrambled-DNA in the presence of p53-WT, suggesting sequence-specific DNA recognition and displacement by p53. Notably, the pattern of RE-binding across the panel was identical between AO-R and EtBr, and similar in Carb-3 (with the exception of pDINP1 and p21, likely associated to the preferential binding observed earlier in Fig. 3) (Fig. 4). Critically, the magnitude of displacement for each RE also correlated with their respective binding affinities towards p53 (Supplementary Table 1), in the order Con-A < p21, RGC < Bax, pDINP1< scram. AO-R, however, provided the best resolution between response elements, showing almost no displacement activity on the scrambled-DNA control sequence (Fig. 4), hence affording a high level of binding discernment.

Direct fluorescence titration of AO-R and AO-C against p21 RE yielded K_d_s per bp of 4.8 ± 0.8 and 12.5 ± 1.0 μM respectively (Supplementary Fig. 5). The determined K_d_ for AO-C is comparable with the ~36 μM value obtained using spectroscopic and isothermal calorimetry methods^39^. Furthermore, the K_d_ of AO-R is highly comparable to the range of values reported for the common FID dyes ethidium bromide and thiazole orange (1.1–15 and 0.3–20 μM respectively)^16,39,40^.

### *In-vitro* applications of AO-R molecular rotor for studying p53-DNA binding

**Figure 5:**
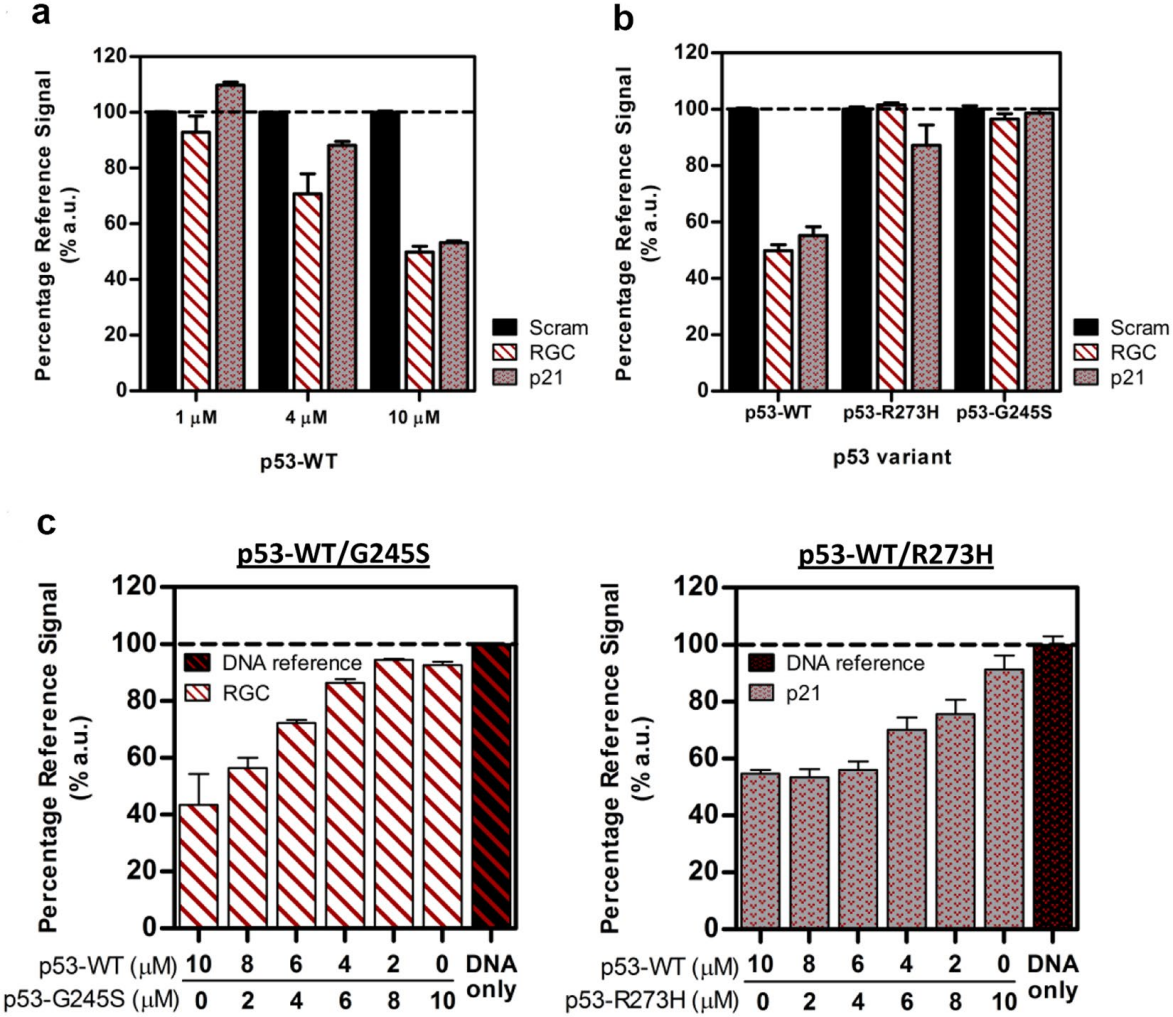
Acridine orange rotor (AO-R) as a fluorescence probe for sequence-specific p53-DNA binding. (**a**) FID assay using purified wildtype p53 protein at indicated concentrations on response elements RGC, p21, and scrambled control DNA (black bars) pre-complexed with AO-R. Data normalized to scram control showing FID effects through sequence-specific DNA binding. (**b**) FID assay using either wildtype p53, p53 G245S mutant or p53 R2733H mutant (10 µM) on DNA response elements pre-mixed with AO-R. (**c**) FID assay using AO-R to examine dominant effects of mutant p53 proteins, which were first prepared to contain different proportions of wildtype and mutant p53 molecules (10 µM final). Left: FID response of AO-R pre-complexed RGC-RE exposed to p53 mixtures containing wildtype and G245S mutant proteins. Right: FID response of AO-R pre-complexed p21-RE exposed to p53 mixtures containing wildtype and R273H mutant proteins. Black bar on right shows RE-AO-R complex reference signal at 100% (black hash line). All data shows mean ± S.D. of 3 individual FID experiments.

Rotor AO-R was selected for further characterization due to its favorable FID properties. The p21 and RGC sequence motifs represent two of the most well-characterized and physiologically relevant p53-binding DNA elements that govern cell fate^41,42^. FID experiments using compound AO-R showed a p53 concentration-dependent binding to both p21 and RGC response elements, but not scrambled DNA control (Fig. 5a). Additionally, DNA-binding was markedly reduced when two cancer-associated inactive p53 mutants (R273H or G245S mutation)^43^ were assayed (Fig. 5b). p53 binds DNA as a homotetramer with momomeric subunits associating through the tetramerization domain. Cells heterozygous (p53^mutant/+^) for the mutant allele (germline or somatic mutated) are predisposed to cellular transformation due in part to diminished p53 activity from dominant-negative effects exerted through the formation of mutant-wildtype hetero-tetramers^44^. The often ensuing loss-of-heterozygosity (LOH) in the remaining functional wildtype allele during cancer development, frequently recapitulated in animal studies^45,46^, alludes to pro-survival advantages provided by p53 mutants. In addition, p53^mutant/+^ cancers exhibit unique tumor spectra that are allele-specific and metastasize more frequently^47,48^, strongly suggesting mutant-dependent dominant gain-of-functions when in the presence of wildtype p53. We investigated mutant and gene-specific dominant effects by studying changes in canonical DNA binding using mutant and wildtype p53 protein mixtures. The p53-G245S mutant protein displayed a strong dominant-negative effect on wildtype p53′s ability to bind the RGC response element (Fig. 5c), where a notable reduction in DNA binding activity was observed in the presence of low mutant concentrations (20% mutant), and completely ablated with 80% mutant protein. Contrastingly, wildtype p53 binding of p21-RE was more recalcitrant to the dominant negative effects of p53-R273H mutant, as binding remained unchanged even in the presence of 40% mutant protein, and was retained considerably even at the highest proportion of mutant protein tested (Fig. 5c).

### Use of AO-R molecular rotor for studying apoptosis in live organisms

**Figure 6:**
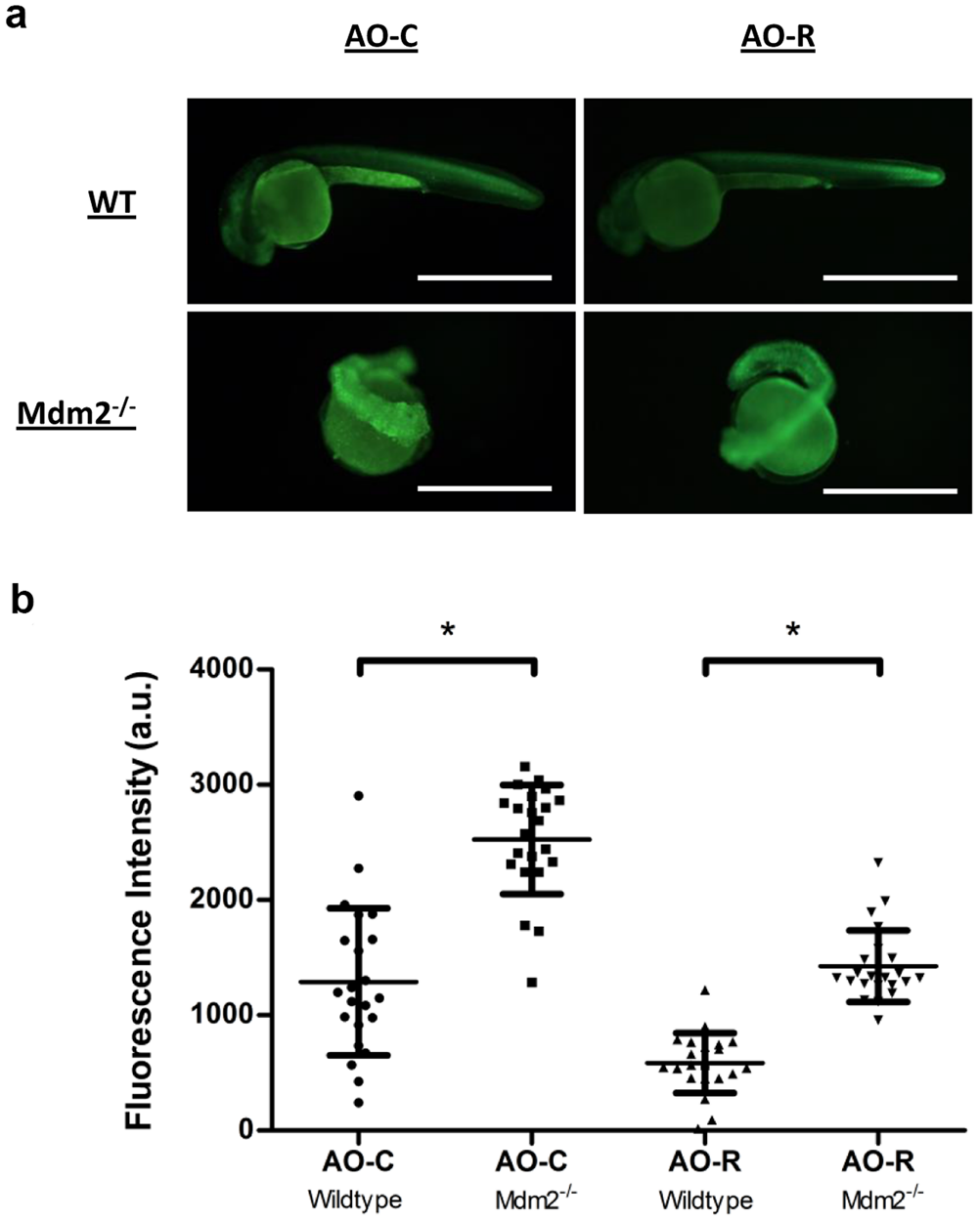
*In-vivo* staining of live zebrafish embryos using compound AO-R. (**a**) Live imaging of either wildtype (top panels), or Mdm^−/−^ (bottom panels) 1 day post fertilization zebrafish embryos stained using either AO-C (left panels) or AO-R (right panels) dyes. Scale bars measure 1 mm. (**b**) Fluorescence intensity of individually measured whole, wildtype or Mdm2 knock-out, zebrafish embryos stained with either AO-C or AO-R dyes. (N = 22; *P < 0.0001, unpaired, two-tailed student *t* test).

Acridine orange is widely used as a highly selective dye in live embryos of zebrafish (*danio rerio*)^49^ and *drosophila melanogaster*^50^, where it strongly labels both the cytoplasm and nucleus of apoptotic cells. This phenomenon, which isn’t observed in live or necrotic cells, is attributed to specific apoptosis-related cellular changes that promote nucleic acid intercalation and staining of condensed apoptotic nuclei^51,52^. Cellular levels of pro-apoptotic p53 are stringently controlled by the E3 ligase Mdm2, deletion of which results in an embryonic lethal phenotype^53^. We therefore compared the fluorescence intensity of AO-C and AO-R on either wildtype or Mdm2^−/−^ zebrafish embryos (generated through incrossing of Mdm2^+/−^ fish), previously reported to stain strongly with acridine orange due to the large number of apoptotic cells that are otherwise absent in wildtype fish^54^. The results, similar to the response seen with DNA intercalation, showed considerably lower levels of background (wildtype embryos) with comparable levels of fluorescence activation (mutant embryos) when using AO-R over AO-C (Fig. 6a). Fluorescence signal of the embryos individually quantified showed ~3-fold reduction in background signal and a 32% increase in turn-on fluorescence (2.5-fold compared to 1.9-fold) for AO-R compared to AO-C. Further experiments are required to understand the improved performance of AO-R in this assay, in particular the signal contribution arising from DNA-binding.

## Discussion

We tested Jul-R, 2 new fluorescent molecular rotors and 3 minor groove binders that possess an intercalating electron-donor moiety in their ability to detect DNA and p53-DNA interactions. The rotors displayed an expected enhancement in fluorescence intensity both in an environment of increasing viscosity, and in the presence of double-stranded DNA. The titration of increasing compound concentration measuring DNA-induced turn-on fluorescence showed that the synthesized rotors were comparable or brighter than several commercial dyes. A screen of the compounds against a panel of DNA response elements suggest that several displayed preferential activities in response to certain DNA sequences, rendering them less suitable for FID assays on proteins with the propensity to target extensive arrays of DNA motifs and sequences (eg. master transcription regulators like p53), but possibly useful for other downstream applications involving decorating or targeting specific DNA motifs. Compounds EtBr, AO-R, and to a lesser extent, Carb-3, were more suitable for the FID assay, and displayed relative p53-dependent displacement activity consistent with published p53-RE binding affinities. The DNA binding affinity of AO-R was also comparable to ethidium bromide and thiazole orange, the most commonly used FID dyes. Along with its relatively lower sequence discrimination, AO-R may find use in screening campaigns interrogating large numbers of variant DNAs. Furthermore, in certain cases the spectral properties of AO-R (Table 1) may be more compatible with assay development, for example when potentially auto-fluorescent small molecules are being evaluated for modulation of p53 activity (e.g. reactivation of non DNA-binding mutant p53).

Modifications of the molecular structure have been carried out to tune the electronic properties of the molecular rotors. Previous studies have shown that replacement of the amino electron donor group with a weaker electron donor results in a blue shift in both the excitation and emission maxima^22^. The longer the conjugation length of the linker moiety, the longer the emission wavelength and the larger the Stokes shift. In addition, replacement of the nitrile electron acceptor moiety with methyl ester or phenyl sulfonyl results in an increase in the emission signals due to the stabilization of the excited state brought about by the change in dipole moment. Molecular rotors based on the aminobenzylidene–cyanoacetamide moiety have also been directly incorporated into DNA using modified nucleosides, with subsequent protein binding resulting in fluorescence turn-on^55^. In our study, AO-R gave the largest signal increase upon DNA binding and provided a high level of sensitivity and precision to p53-DNA interaction in a sequence-dependent manner. The difference between AO-R and commercial acridine orange is the addition of a (2-carboxy-2-cyano) vinyl moiety in AO-R. This endows the molecule its twisted intercalating charge transfer properties. The AO-R rotor also has a lower background than commercial acridine orange because of an additional non-radiative relaxation pathway when in free solution. Excited state energy can be dissipated through twisted intramolecular charge transfer via the rotation of this vinyl bond. This also contributes to its increased sensitivity to binding interactions and greater increase in fluorescence compared to acridine orange.

AO-R enabled discrimination between wild type and mutant p53 DNA binding in the facile and homogenous FID assay. The assay measured gradated inhibition of wt-p53 in the presence of increasing amounts of two common mutant p53 variants, suggesting utility in small-molecule mutant p53 reactivation studies/screening campaigns, where a reciprocal phenotype is expected. The assay will be useful for higher throughput combinatorial sampling of the dominant-negative activity of cancer-associated p53 mutants across diverse panels of response elements, and to investigate how interactions of wild-type/mutant p53 with other p53 family members (p63 and p73)^56^ impacts DNA binding. Furthermore, it may fuel identification of novel therapeutically relevant genes or protein targets, especially when used in combination with fluorescence correlation spectroscopy (FCS) that allows simultaneous inspection of p53 oligomerization and DNA-binding kinetics^57^.

Further studies to both delineate the precise binding mode(s) of AO-R with DNA and assess its applicability to other DNA binding proteins are required to fully benchmark this new probe against the robust and commonly used FID dyes EtBr and thiazole orange.

## Materials and Methods

### Molecular rotor synthesis

Syntheses of Compounds I, V and VI were carried out as previously reported^31,58^.

### Preparation of Py-R (II)

Pyrenecarboxaldehyde and cyanoacetic acid was weighed into a 25 mL round-bottom flask flushed with argon. Triethylamine was then added to the reaction mixture after solvating in anhydrous THF. The reaction mixture was then heated to 55 °C overnight, then evaporated to dryness and purified via column chromatography to yield pale orange solids.

^1^H NMR (DMSO, 400 MHz) *δ*8.14 (t, *J* = 7.6 Hz, 1H), 8.23 (d, 1H, *J* = 8.9 Hz), 8.30 (d, 1H, *J* = 8.7 Hz), 8.34 (d, 1H, *J* = 7.5 Hz), 8.39 (m, 3H), 8.57 (d, 1H, *J* = 8.1 Hz), 9.00 (s, 1H). ^13^C NMR (100 MHz, DMSO) *δ* 162.8, 145.8, 132.2, 130.7, 130.1, 129.1, 128.8, 127.9, 127.1, 126.7, 126.3, 126.1, 125.8, 124.9, 123.8, 123.6, 122.6, 120.4, 118.9.

### Preparation of AO-R (III)

Acridine Orange aldehyde (0.12 g, 0.42 mmol, 1 eq.) and cyanoacetic acid (0.053 g, 0.63 mmol, 1.5 eq.) was weighed into a 25 mL round-bottom flask flushed with argon. Triethylamine (0.29 mL, 2.09 mmol) was then added to the reaction mixture after solvating in anhydrous DMF. The reaction mixture was heated to 55 °C overnight, then evaporated to dryness and purified via column chromatography to yield orange solids (1.69 mg, 1%).

^1^H NMR (CDCl_3_, 400 MHz) *δ* 1.26 (s, 3H), 3.05 (s, 3H), 3.24 (s, 6H), 7.06 (dd, 1H, *J* = 2.4, 9.3 Hz), 7.31 (s, 1H), 7.47 (s, 1H), 7.60 (d, 1H, *J* = 9.0 Hz), 7.69 (d, 1H, *J* = 9.4 Hz), 8.36 (s, 1H). ^13^C NMR (100 MHz, CDCl_3_) *δ* 154.8, 154.6, 143.7, 142.8, 141.5, 130.4, 130.3, 117.8, 117.2, 115.2, 95.4, 93.8, 40.6, 30.4, 29.9.

### Preparation of 3-methyl-2-(methylthio)benzo[*d*]thiazol-3-ium 4-methylbenzenesulfonate (12)

2-methylthiobenzothiazole (5.00 g, 27.62 mmol, 1 eq.) was weighed into a 25 mL round bottom flask under argon. Methyl p-toluenesulfonate (4.58 mL, 30.39 mmol, 1.1 eq.) was then added and the reaction mixture was allowed to stir at 130 °C for 1 hour. Acetone was then added after cooling to 70 °C until white precipitate appeared. The mixture was then refluxed for another 30 minutes before cooling to room temperature. Precipitate was collected by filtration and dried to yield pale yellow solids (10.02 g, 99%).

^1^H NMR (CH_3_OD, 400 MHz) *δ* 2.35 (s, 3H), 3.12 (s, 3H), 4.15 (s, 3H), 7.19 (d, 2H, *J* = 4.0 Hz), 7.67 (d, 2H, *J* = 8.0 Hz), 7.73 (t, 1H, *J* = 8.0 Hz), 7.85 (t, 1H, *J* = 8.0 Hz), 8.07 (d, 1H, *J* = 8.0 Hz), 8.22 (d, 1H, *J* = 8.0 Hz). ^13^C NMR (CH_3_OD, 100 MHz) *δ* 183.3, 144.2, 143.7, 141.6, 130.8, 130.0, 129.8, 128.6, 127.0, 124.7, 116.5, 36.9, 21.3, 18.5.

### Preparation of 1-(3-aminopropyl)-4-methylquinolin-1-ium bromide (13)

3-bromopropylamine hydrobromide (6.06 g, 27.68 mmol, 1.46 eq.) was weighed into an argon-flushed 25 mL round-bottom flask. Ethanol (5 mL) was then added to dissolve. Upon addition of Lepidine (2.5 mL, 18.91 mmol, 1 eq.), the reaction mixture was heated to 40 °C overnight. Pale pink precipitate (1.67 g, 32%) formed was filtered, washed and dried.

^1^H NMR (D_2_O, 400 MHz) *δ* 2.55 (quin, 2H, *J* = 8.0 Hz), 3.10 (s, 3H), 3.29 (t, 2H, *J* = 8.0 Hz), 5.16 (t, 2H, *J* = 8.0 Hz), 7.98 (d, 1H, *J* = 4.0 Hz), 8.11 (t, 1H, *J* = 4.0 Hz), 8.32 (t, 1H, *J* = 8.0 Hz), 8.46 (d, 1H, *J* = 8.0 Hz), 8.60 (d, 1H, *J* = 8.0 Hz). ^13^C NMR (100 MHz, D_2_O) *δ* 160.6, 147.3, 137.2, 135.6, 129.8, 127.3, 122.6, 118.2, 54.3, 36.5, 27.0 19.6.

### Preparation of TO (IV)

3-methyl-2-(methylthio)benzo[*d*]thiazol-3-ium 4-methylbenzenesulfonate (1.05 g, 2.86 mmol, 1 eq.) and 1-(3-aminopropyl)-4-methylquinolin-1-ium bromide (0.8 g, 2.86 mmol, 1 eq.) was weighed into 25 mL round-bottom flask under argon. 30 mL of ethanol was added to dissolve. Triethylamine (0.8 mL) was then added and the reaction was stirred at room temperature for 1 hour. Red precipitate (0.16 g, 11%) formed upon addition of ether was filtered, washed and dried.

^1^H NMR (CH_3_OD, 400 MHz) *δ* 1.21 (t, 1H, *J* = 8.0 Hz), 2.31 (td, 2H, *J* = Hz), 3.00 (s, 3H), 3.18 (m, 3H), 3.88 (s, 3H), 6.70 (t, 1H, *J* = 8.0 Hz), 6.74 (s, 1H), 6.77 (d, 1H, *J* = 8.0 Hz), 6.90 (td, 1H, *J* = 4.0, 8.0 Hz), 7.14 (d, 1H, *J* = 8.0 Hz), 7.23 (dd, 1H, *J* = 4.0, 8.0 Hz), 7.32 (t, 1H, *J* = 8.0 Hz), 7.50–7.71 (m, 4H), 7.88 (t, 1H, *J* = 8.0 Hz), 8.08 (d, 1H, *J* = 8.0 Hz), 8.29 (d, 1H, *J* = 8.0 Hz), 8.54 (d, 1H, *J* = 8.0 Hz). ^13^C NMR (100 MHz, CH_3_OD) *δ* 162.1, 158.4, 150.8, 145.3, 142.2, 141.9, 139.1, 134.3, 129.4, 128.1, 127.5, 126.5, 123.7, 123.3, 122.1, 119.4, 113.6, 110.0, 109.5, 89.1, 55.2, 52.3, 34.0, 30.5, 30.2.

### Viscosity experiments

The rotors were dissolved in spectroscopy-grade DMSO to obtain a concentrated stock solution of 100 mM. The solution was vortexed to ensure complete dissolution. For each rotor, 6 µL of the 100 mM stock was dissolved in 1200 µL of ethylene glycol in each 1.5 mL tube. 3 mL of glycerol was then heated in a boiling bath to reduce viscosity and improve pipetting. In 5 separate tubes, add 200 µL of stained ethylene glycol (i.e. with respective rotor added) to 800 µL of unstained ethylene glycol; 200 µL of stained ethylene glycol, 600 µL of unstained ethylene glycol to 200 µL glycerol; 200 µL of stained ethylene glycol, 400 µL of unstained ethylene glycol to 400 µL of glycerol; 200 µL of stained ethylene glycol, 200 µL of unstained ethylene glycol to 600 µL glycerol; and 200 µL of stained ethylene glycol to 800 µL glycerol. The fluids will then have a glycerol content of 0%, 20%, 40%, 60%, 80% respectively. The five tubes were then placed on an inverting mixer and allowed to mix for at least 2 hours while the temperature equilibrate to room temperature. Measurements were taken using 96-well Corning Flat-bottom Black plate and Tecan Infinite M-1000 Spectrophotometer. All samples have a final rotor concentration of 100 µM.

### Experimental for characterization of rotor-DNA binding

Mixtures containing specified DNA and rotor concentrations were set-up in 50 μL reaction volumes consisting of 5% (v/v) DMSO, 80% (v/v) p53 binding buffer (25 mM NaPi, pH 7.2, 150 mM KCl, 4 mM DTT), 15% (v/v) DNA buffer (10 mM Tris-HCl, pH 8.0, 50 mM NaCl). Rotor-DNA mixtures were constituted in 96-well plates (round-bottom black plate, Corning), and incubated in the dark at room temperature for 20 mins before fluorescence intensity was measured using the EnVision Multilabel Plate Reader (PerkinElmer) following Ex/Em wavelengths specified in Table 1. Scrambled-DNA (scram) double-stranded oligonucleotide molecules are used in experiments for evaluating compound-DNA binding. Measurement parameters used were: excitation light, 100%; detector gain, 500; no. flashes, 200. Figures are plotted using raw fluorescence signals from triplicate experimental repeats where no data manipulation/transformation was performed (error = ±standard deviation). To generate plot for DNA-dependent turn-on fluorescence (Fig. 2H), ‘dye-only’ background signal were subtracted from total fluorescence signal, raw data prior to background subtraction are shown in Supplementary Fig. 3. For Jul-R and Py-R readings were also taken at maximum measurement configuration (excitation light, 100%, detector gain, 1000, no. flashes, 5000). For consistency, compounds were always pre-diluted in 100% DMSO solution. Rotor-DNA binding heat map was generated by measuring fluorescence signal from 4 μM of each fluorogen alone, or in the presence of 250 nM of dsDNA oligonucleotide from a panel of unique sequences. For each series (specific compounds), fluorescence turn-on is shown on a color-represented scale where the lower limit (violet) is self-referenced to respective background signals (no DNA) and the upper limit (red) to the maximum gain observed in the entire experiment (10 000 a.u.), allowing a standardized frame of comparison for turn-on fluorescence across different compounds. Double-stranded DNA response elements (DNA-RE) were made by mixing equi-molar concentrations of complimentary ssDNA oligomers (IDT, Singapore) in DNA buffer (10 mM Tris-Cl pH 7.5, 50 mM NaCl) and heated at 95 °C for 5 minutes before slowly cooling to room temperature. DNA oligonucleotide sequences can be found in Supplementary Figures, Table 1. Direct fluorescence titration of AO-R and AO-C versus a hairpin p21 RE (5′-GAAGAAGACTGGGCATGTCTAAAAAAGACATGCCCAGTCTTCTTC-3′) was carried out in 1 mL cuvettes comprising 80% (v/v) p53 binding buffer (25 mM NaPi, pH 7.2, 150 mM KCl and 4 mM DTT), 15% (v/v) DNA buffer (10 mM Tris-HCl, pH 8.0, 50 mM NaCl) and AO-R/AO-C (100 nM). p21-RE (50 μM) was added in 2 μL aliquots and fluorescence measured after 5 min incubation at room temperature. Addition of DNA continued until system reached saturation and fluorescence remained constant. Titrations were carried out in duplicate with measurement parameters set to: excitation light, 100%; detector gain, 500; no. flashes, 200. The molar fluorescence of free dye was determined by measuring the fluorescence of increasing concentrations of each dye (in the absence of DNA) and using linear regression to find the gradient. This value was used as described^59^ for determination of approximate K_d_s derived by least squares fitting of data using using GraphPad Prism version 7.03 (GraphPad Software, La, Jolla California USA).

### Expression and purification of p53 protein

Recombinant p53 proteins (residues 94–360, carrying N-terminal hexa-histidine tag) were expressed in Rosetta (DE3)-T1R cells from the pNIC28-Bsa4 vector. Expression cultures supplemented with 100 μM ZnSO_4_ were induced with 0.5 mM IPTG (OD_600_) and grown overnight at 18 °C, before cells are collected and resuspended in lysis buffer (100 mM HEPES pH 8.0, 500 mM NaCl, 0.5 mM TCEP, 10 mM Imidazole, 10% (v/v) glycerol, with Benzonase endonuclease (Merck) and Protease Inhibitor Cocktail Set III, EDTA free (Calbiochem)) the following day. Cells were lysed on ice by sonication and supernatants were first filtered (1.2 μm, Sartorius) and affinity purified through a Ni-NTA column (AKTA Express, GE Healthcare). Columns were washed with 20-column volumes of wash buffer 1 (20 mM HEPES pH 7.5, 500 mM NaCl, 0.5 mM TCEP, 10 mM Imidazole, 10% (v/v) glycerol), 20-column volumes of wash buffer 2 (20 mM HEPES pH 7.5, 500 mM NaCl, 0.5 mM TCEP, 25 mM imidazole, 10% (v/v) glycerol) before proteins were eluted with 20 mM HEPES pH 7.5, 500 mM NaCl, 0.5 mM TCEP, 500 mM Imidazole, 10% (v/v) glycerol, and further passed through a gel filtration column (HiLoad 16/60 Superdex 200 prep, GE healthcare). Eluted fractions were SDS-PAGE analysed and further concentrated (10 kDa Amicon ultra-15 centrifugal filter units, Millipore) before buffer exchange into p53 binding buffer 25 mM NaPi, pH 7.2, 150 mM NaCl and 4 mM DTT (Zeba desalting spin columns, Thermo Fisher Scientific). Protein identity was additionally mass spectrometry verified.

### Experimental for p53-DNA Nbinding assay

p53-DNA binding reaction mixtures were prepared by first mixing DNA-REs with fluorogenic compounds for 20 minutes before p53 protein is added. After an additional 20 minutes, fluorescence intensity was measured on the EnVision Multilabel Plate Reader (PerkinElmer). To account for slight signal variabilities between wells, fluorescence turn-on signal from DNA-rotor interaction was first quantified, before the addition of proteins, and used for signal normalization within each experimental set. Each FID assay data point was obtained by measuring in replicates, differences in fluorescence intensity between samples with or without p53 proteins. Binding reactions were prepared to contain 1μM DNA response element and 10 μM rotor compounds in 50 μL reaction volumes with or without p53 proteins. Final reaction mixtures consist of 5% (v/v) DMSO, 80% (v/v) p53 binding buffer (25 mM NaPi, pH 7.2, 150 mM KCl and 4 mM DTT) and 15% (v/v) DNA buffer (10 mM Tris-HCl, pH 8.0, 50 mM NaCl).

### Staining and imaging of zebrMafish embryos

Zebrafish embryos collected at 1-day post fertilisation were incubated with 0.1% acridine orange solution in egg water (0.3% w/v sea-salt solution) for 30 minutes at room temperature before washing in fresh egg water 5 times for 10 minutes each. The embryos were imaged under a green fluorescent filter on the Leica M165FC fluorescence stereomicroscope (Leica, Solms, Germany). Individual embryos were then transferred onto a 96-well plate and quantified using the EnVision Multilabel Plate Reader (PerkinElmer). Data shown are background subtracted using egg water-only values from respective samples. All experiments were conducted in accordance to the ethical guidelines and approved by the Institutional Animal Care and Use Committee, Biomedical Research Council.

## Electronic supplementary material

Supporting Information

## Data Availability

The datasets generated during and/or analysed during the current study are available from the corresponding authors on reasonable request.
