# Supplementary material for: A novel molecular rotor facilitates detection of p53-DNA interactions using the Fluorescent Intercalator Displacement Assay
Source: Sci Rep. 2018 Aug 28;8:12946. doi: 10.1038/s41598-018-31197-9 (PMC6113202; doi:10.1038/s41598-018-31197-9)
Supplement: Supplementary file 1 — Supporting Information [file 41598_2018_31197_MOESM1_ESM.docx]

**Supporting Information:**

**A novel molecular rotor facilitates detection of p53-DNA interactions using the Fluorescent Intercalator Displacement Assay**

Walter L. Goh^2,#^, Min Yen Lee^1,#^, Lim Ting Xiang^2^, Joy S. Chua^2^, Sydney Brenner^1^, Farid J. Ghadessy^2*^ and Yin Nah Teo^1,3^

^1^ Molecular Engineering Lab, Biomedical Sciences Institutes, A*STAR, 61 Biopolis Drive, Singapore 138673

^2^ p53 Laboratory, A*STAR, 8A Biomedical Grove, #06-06 Immunos, Singapore 138648

^3^ Division of Chemistry and Biological Chemistry, SPMS, Nanyang Technological University, Singapore 637371


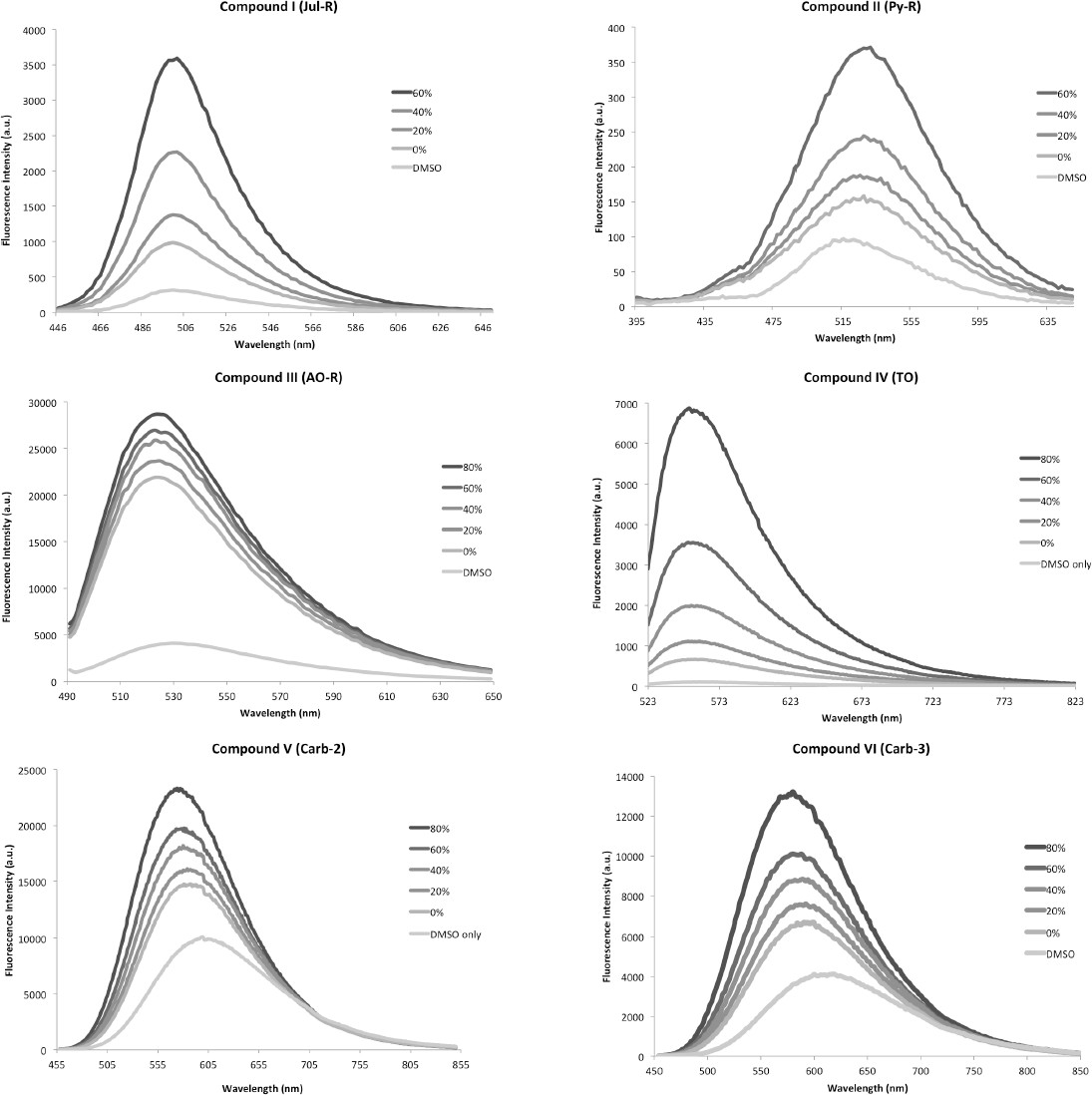


**Supplementary Figure 1**. Emission spectra of rotors in increasing viscosity. % indicate percentages of glycerol in ethylene glycol. Final rotor concentration is 10 µM.

**Supplementary Figure 2**. Fluorescence activity of **Py-R** (100nM) with increasing concentrations of double stranded DNA-oligo at Ex=342nM. Error bar shows S.D. of triplicate readings.


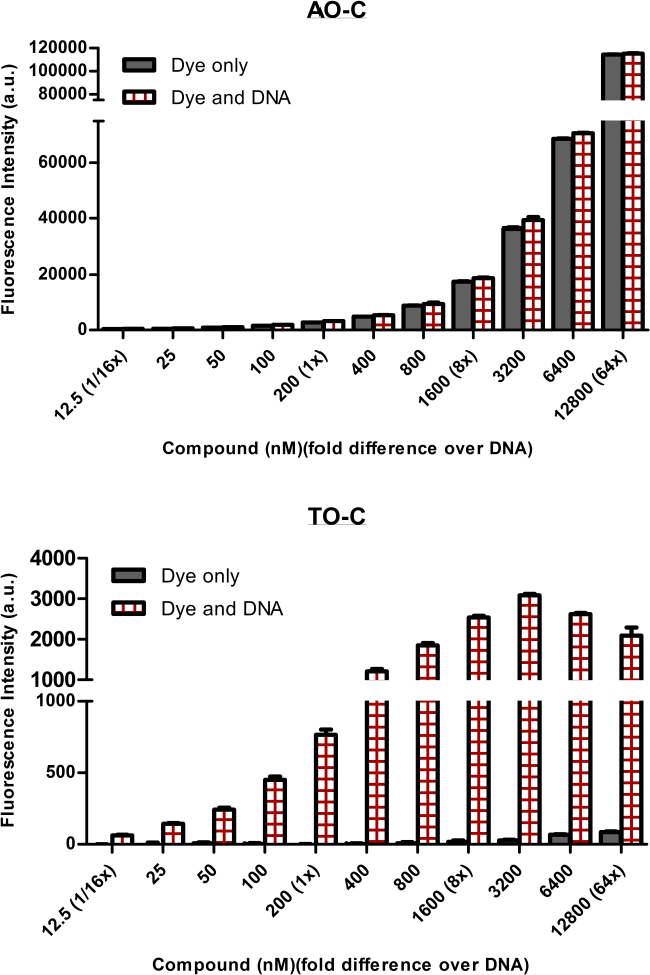

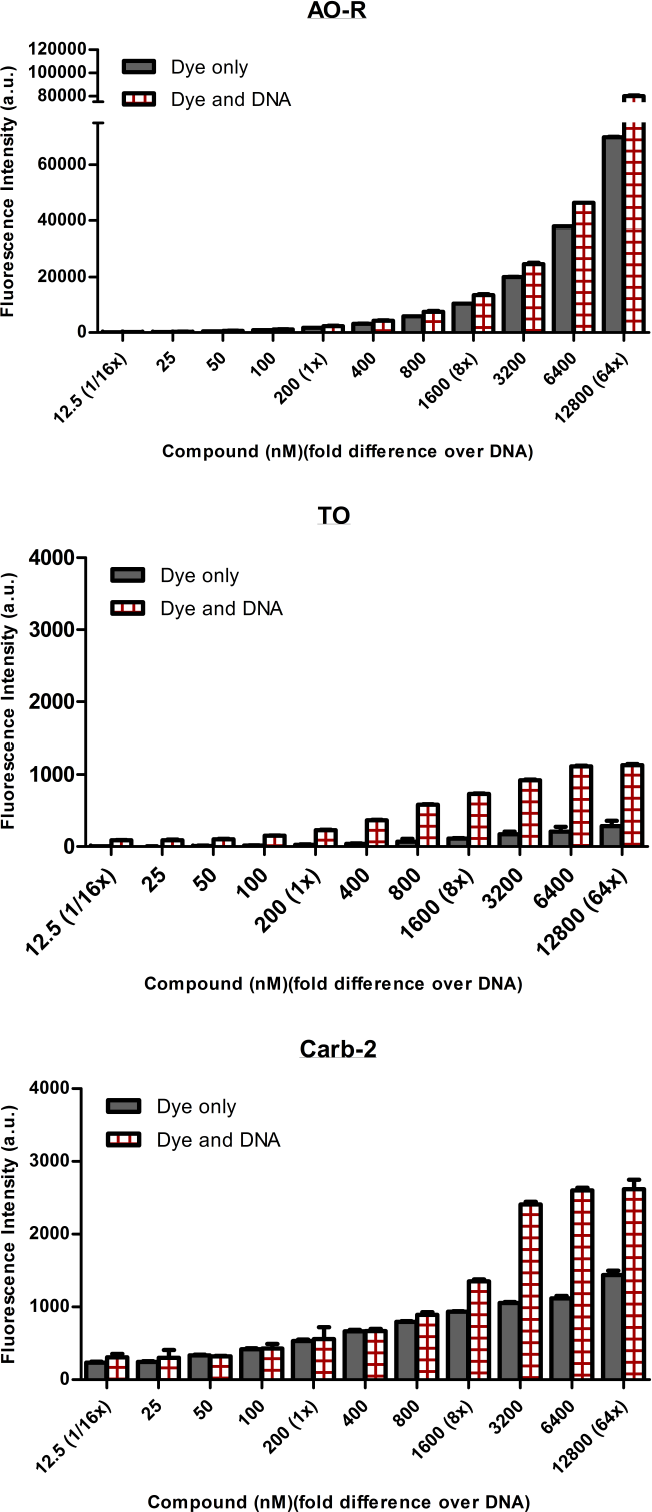

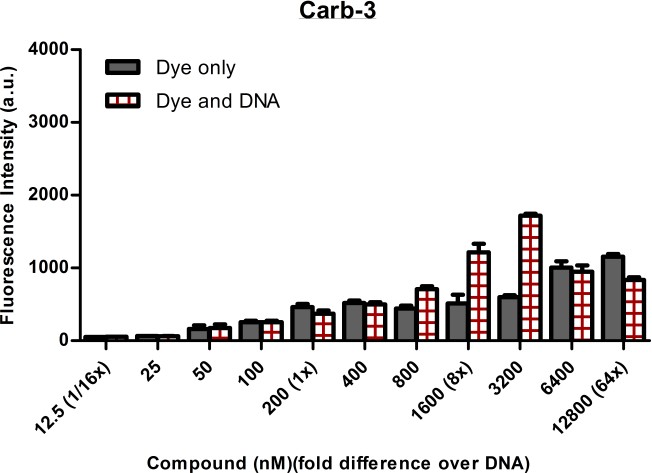

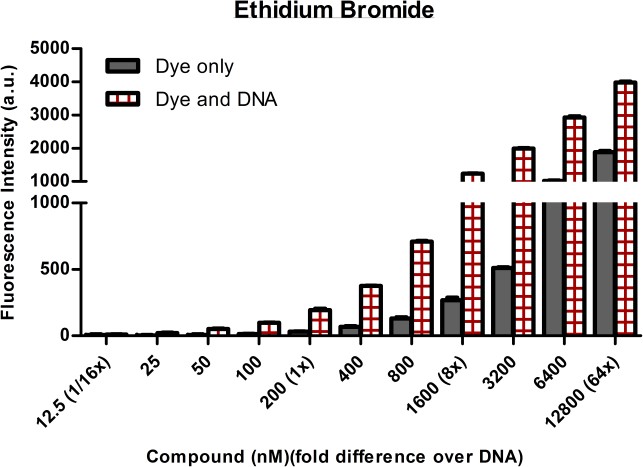


**Supplementary Figure 3**. Fluorescence intensities of rotor compounds (0.0125 - 12.8 uM) either alone (grey bars) or in presence of 30 bp DNA oligomer (200 nM) (red checkered bars). Error bar shows S.D. of triplicate readings.

## 2200

**2000**

**Fluorescence (RFU)**

**1800**

**1600**

**1400**

**1200**

**1000**

**800**

**600**

**400**

**200**

**0**

**0 10 20 30 40 50 60**

**Time (mins)**

**100**

## 80


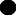


TO-C

TO

**Percentage of max TO-C fluorescence (%)**

**60**

**40**

**20**

**0**

**Supplementary Figure 4**. Time-lapse measurement of fluorescence signal from mixture containing 100 nM of either thiazole orange compounds (TO and TO-C) and 5µM DNA (30-base pair DNA oligomer). Error bar shows S.D. of triplicate readings.


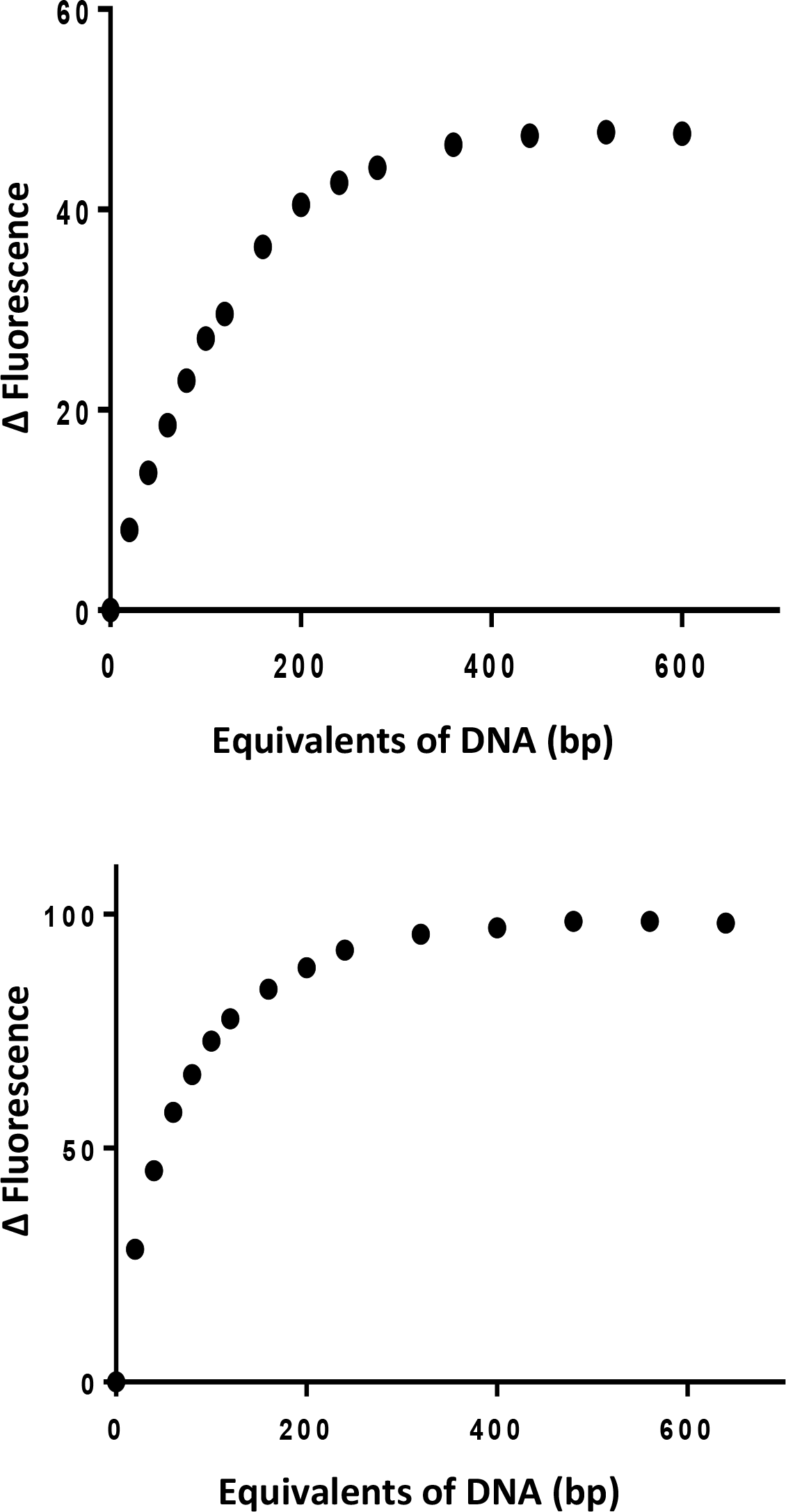
**a**

**b**

**Supplementary Figure 5**. A. Titration of p21-RE hairpin DNA versus AO-C (100 nM). B. Titration of p21-RE hairpin DNA versus AO-R (100 nM).

| **DNA-RE** | **DNA sequence (5’ to 3’)** | **Length (bp)** | **Reported K_D_ (nM) (^1, 2^)** |
| --- | --- | --- | --- |
| Scram | GTCGTTCTGA TCCAGGTGAT GTAGTCGACG | 30 | - |
| Con-A | GGGCATGTCC GGGCATGTCC | 20 | 0.5 |
| PUMA | CGCGCCTGCA AGTCCTGACT TGTCCGCGGC | 30 | 7.1 |
| p21 | TAGAGGAAGA AGACTGGGCA TGTCTGGGCA | 30 | 4.9 |
| Noxa | ATCTGAGGCT TGCCCCGGCA AGTTGCGCTC | 30 | 8.6 |
| Bax | TGGGCTCACAA GTTAGAGACA AGCCTGGGC | 30 | 73 |
| RGC | CACATGCCTT GCCTGGACTT GCCT | 24 | 6 |
| pDINP1 | TTATAGAACT TGGGGGAACA TGTTTACCAA | 30 | 98 |
| IGF-BP3 | CTAGAAAACA AGCCACCAAC ATGCTTGCAT G | 31 | 81 |

**Supplementary Table 1.** p53 response elements and control DNA sequences

## References

1. 1. Weinberg, R.L., Veprintsev, D.B., Bycroft, M. & Fersht, A.R. Comparative binding of p53 to its promoter and DNA recognition elements. *J Mol Biol* **348**, 589-596 (2005).
2. 2. Balagurumoorthy, P. et al. Four p53 DNA-binding domain peptides bind natural p53-response elements and bend the DNA. *Proc Natl Acad Sci U S A* **92**, 8591-8595 (1995).
